# Supplementary material for: A Dedicated Mycosis Flask Increases the Likelihood of Identifying Candidemia Sepsis
Source: J Fungi (Basel). 2023 Apr 4;9(4):441. doi: 10.3390/jof9040441 (PMC10146598; doi:10.3390/jof9040441)
Supplement: Supplementary file 1 [file jof-09-00441-s001.zip › jof-2307301-supplementary.pdf]

Table S1: Blood culture system used by the different hospitals and departments in 2018 and 2020

**Department of clinical microbiology**	**Hospitals serviced in 2018, blood culture system***	**Hospitals serviced in 2020, blood culture system****
Rigshospitalet	Rigshospitalet, BACTEC	Rigshospitalet, BACTEC
Herlev	Gentofte Hospital, BACTEC<br>Herlev Hospital, BACTEC<br>Nordsjællands hospitaler, BacTAlert	Gentofte Hospital, BACTEC<br>Herlev Hospital, BACTEC<br>Nordsjællands hospitaler, BACTEC
Hvidovre	Amager Hospital, BacTAlert<br>Bispebjerg Hospital, BacTAlert<br>Bornholm sygehus, BacTAlert<br>Frederiksberg Hospital, BacTAlert<br>Glostrup Hospital, BacTAlert<br>Hvidovre Hospital, BacTAlert	Amager Hospital, BACTEC<br>Bispebjerg Hospital, BACTEC<br>Bornholm sygehus, BACTEC<br>Frederiksberg Hospital, BACTEC<br>Glostrup Hospital, BACTEC<br>Hvidovre Hospital, BACTEC

* Standard blood culture set consisted of two aerobic and two anaerobic flasks
** Standard blood culture set consisicted of two aerob, one anaerobic and one mycosis flask
